# Asylum-seeker women: Coping strategies and mental wellbeing

**DOI:** 10.1177/00207640241291498

**Published:** 2024-10-18

**Authors:** Sara Shishehgar, Leila Gholizadeh, Michelle DiGiacomo, Patricia Mary Davidson

**Affiliations:** 1 Western Sydney University, School of Nursing and Midwifery, NSW, Australia; 2 University of Technology Sydney, NSW, Australia; 3 Faculty of Health, University of Technology Sydney, NSW, Australia; 4 University of Wollongong, NSW, Australia

**Keywords:** Asylum seeker, coping strategies, mantal health, psychological, qualitative research, resilience

## Abstract

**Background:**

Asylum seekers in Australia are subjected to various punitive measures that can affect their psychological wellbeing. The capacity of asylum seekers to adapt and cultivate effective coping strategies can enhance their resilience, facilitate their settlement processes and promote their overall mental health.

**Aim:**

This study aims to explore the coping strategies employed by women who are asylum-seekersthat have the potential to enhance their resilience post migration.

**Method:**

A semi-structured qualitative study was conducted with asylum-seeker women from Iran. Data were analysed using an inductive thematic analysis.

**Results:**

Seventeen participants described their experiences of applying various strategies to enhance their coping ability, resilience and maintain their mental wellbeing when facing ongoing challenges. Problem-solving strategies included social engagement, adjusting life plans and seeking support from formal and informal resources. Emotion-focussed strategies were positive thinking and maintaining hope, avoidance and spirituality. While emotion-focussed strategies enabled the women to manage their stresses temporarily, problem-solving strategies allowed them to effectively address the challenges they faced after migration.

**Conclusion:**

Providing supportive resources for asylum seekers, removing stigma and developing community ties may assist individuals in improving their coping skills, resilience and mental wellbeing.

## Introduction

Forced migration is increasingly driven by social, political and economic factors. The number of forcibly migrant increased to 117.3 million people in 2023 (United Nations High Commissioner for Refugees [UNHCR], 2024a). Of this number, 37.6 million are recognised as refugees, although 6.9 million are still asylum seekers. The number of asylum seekers whose claims for refugee status is still under review increased by about four times over the last 10 years (UNHCR, 2024a). This increasing trend is expected to continue because of progressive unrest, war, violence and injustice (Qi & Bircan, 2023).

Australia has always been considered as a destination for forced migrants. The Department of Home Affairs (2024) reported the number of asylum seekers who arrived in Australia by boat and hold a bridging visa-E (BVE) or waiting for a BVE at 6,192 people in March 2024, of which 2,410 are from Iran. While living in the community, they are often introduced as ‘queue jumpers’ or ‘illegal immigrants’ in government documents and media (Martin, 2021). They are also entitled to punitive measures such as indefinite insecure residency, waiting over a decade for their protection application to be reviewed, limited access to tertiary education and inability to reunite with their immediate family members (UNHCR, 2024b). Asylum seekers have already endured traumatic events before or during migration which resulted in diagnosed mental disorders, including depression, anxiety and post-traumatic stress disorders (PTSD). The experience of stigma and punitive measures may further erode their mental wellbeing (UNHCR, 2024b). While some individuals normalised psychological issues because of migration-related trauma, others developed coping methods and strategies to enhance resilience and maintain their mental wellbeing (Abraham, Lien, & Hanssen, 2018).

Resilience is defined as an individual’s ability to cope with challenges, which itself can be determined by coping strategies that are used by everyone (Karaman et al., 2023). Hosseini et al. (2017) recommended infrastructural strategies including reducing discrimination and providing equal employment opportunities for immigrants to enhance their resilience and mental health. However, other studies believe that individuals should be equipped with coping strategies according to their own strengths and resources to build up resilience (Solberg et al., 2023; Tippens et al., 2021).

Continuing experience of trauma and displacement hurts individuals’ psychological health. This study aims to explore strategies that Iranian asylum-seeker women use to improve their resilience and mental wellbeing post migration.

## Literature review

It is well documented in the literature that forced migrants endure high level of stress post migration due to insecure residency and the experience of relocation. Coping, however, was found as an approach, skill or ability of individuals to manage stressors and prevent or minimise mental health issues. Hocking (2021) explored protective factors as hope, religion and social connectivity that asylum seekers used to buffer sense of loss, uncertainty, loneliness, helplessness and worry. A qualitative study by Ziersch et al. (2020) explored discrimination as a harmful experience that asylum seekers in South Australia may face during the years of settlement in the community. Ignoring and downplaying discrimination, framing discrimination as universal but not unique to Australia, removing visible signs of specific belief or identity like hijab, avoiding social gatherings, choosing a Western name and living in areas occupied with minority populations were outlined as the strategies to respond to discrimination and protect their mental wellbeing (Ziersch et al., 2020).

Hartley et al. (2017) state engagement in physical activity is a coping strategy for asylum seekers who live in Australia for a protracted period of uncertainty. While some participants found physical activity as an effective strategy to reduce their stress, others perceived it difficult to engage in physical activities while they are deprived of basic rights like the right to work (Hartley et al., 2017). The study suggests engaging in physical activity as a strategy that should be coupled with other strategies or supports to be effective in enhancing asylum seekers’ mental health. The supports include but are not limited to lifting financial barriers to access physical activities like welfare support and amendment of immigration policies against asylum seekers (Hartley et al., 2017).

Insecure residency attached to asylum status has been discussed in Australian literature (Ratnamohan et al., 2023; Shishehgar et al., 2023; Van Kooy & Bowman, 2019). They discussed psychological impacts of precarity resulting from insecure residency and strategies asylum seekers may employ to buffer restrictive immigration policies in Australia. Deliberately planning and daily decisions were discussed as effective tactics, are influenced by social and political structure, economic situation and individual responsibilities and skills though (Van Kooy & Bowman, 2019). The authors recruited participants through an organisation that would offer various services to asylum seekers, such as job-seeking assistance. Therefore, asylum seekers with more restrictions such as lack of work permit, might be excluded from the study (Van Kooy & Bowman, 2019).

A recent study by Solberg et al. (2023) found out a positive association between both problem-solving and emotion-focussed coping strategies and reduced rate of anxiety and depression in asylum seekers. This is while that the association does not reflect sense of psychological wellbeing in the same population.

## Method

A narrative approach was conducted to enable a mutual conversation around strategies that Iranian asylum-seeker women employed to build their resilience towards post-migration challenges (Khwaja & Mahoney, 2019; Yates & Leggett, 2016).

## Ethical considerations

Ethics approval was obtained from the University of Technology SydneyHuman Research Ethics Committee (UTS HREC REF NO. 2014000363). To protect the participants’ confidentiality, pseudonyms replaced the names of the participants and all identifying information was removed from the transcripts. Obtaining verbal consent reassured participants about the confidentiality of their sensitive data and identity (Killawi et al., 2014).

## Sampling and recruitment

A location sampling was used to access Iranian asylum-seeker women as a hard-to-reach population (Reichel & Morales, 2017). After obtaining permission from the multicultural community centre in Western Sydney, the first author attended their weekly gatherings for a period of 6 months and introduced the study to the asylum seekers. The prolonged engagement with the participants prior to the interviews created trust and rapport (Tatah, 2016). Those women who would hold BVE, speak Farsi and have lived in Australia for 2 to 3 years were invited to participate in the study. To avoid exacerbation of psychological conditions due to recalling traumatic events, the researcher excluded women who mentioned being diagnosed with severe depression and Post Traumatic Stress Disorders (PTSD; McVane, 2020).

In addition, snowball sampling was used to increase the representativeness of the sample via increasing access to socially isolated women (Marcus et al., 2017).

## Data collection

Semi-structured and face-to-face interviews were conducted in the participants’ homes using a socio-demographic questionnaire and an interview guide by the first author who is a native Farsi speaker. The interview guide was developed based on literature review, research questions and objectives of this study (Roberts, 2020). The interview guide was reviewed following the first interview to ensure the questions were clear.

To enhance credibility and rigour of the collected data the researcher conducted an in-situ member checking to seek clarification from the participants whenever the answers were not clear (Birt et al., 2016). The researcher considered a two-week distance between two interviews to have adequate time to reflect on her own emotions through writing a journal entry, debriefing with the co-researchers and seeking emotional support if required. As per the ethics approval for this study, the researcher was not allowed to follow up with the participants after the interviews.

The first researcher conducted and transcribed the interviews in Farsi, then translated them into English which were back-translated by another researcher who was fluent in English and Farsi to increase the accuracy of the transcripts. Interviews were audio recorded and continued until data saturation was reached (Fusch & Ness, 2015).

## Data analysis

A thematic analysis was undertaken to create themes and sub-themes of the interviews (Harding, 2018). First, the researcher conducted a close reading of the transcripts and developed summary tables in excel. Second, three researchers coded the transcripts independently to build preliminary concepts and supported each code with examples of excerpts. Third, the researchers discussed codes in two meetings and merged similar codes into one category. Next, the first researcher reviewed the emerged categories and developed preliminary themes and sub-themes. Lastly, all researchers discussed the emerging themes and sub-themes and renamed them to ensure they appropriately reflect their contents (Harding, 2018). All above steps which are aligned with peer examination enhanced credibility and ensured validity of this qualitative study findings (Thomas & Magilvy, 2011).

## Results

A total of 17 participants (Table 1) talked about problem-solving and emotion-focussed strategies they applied to maintain their resilience and psychological wellbeing. Problem-solving strategies like social engagement, life plans adjustment and seeking support from formal and informal resources consolidated the women’s strength to tackle language barriers, acculturate and become productive and valuable persons in the new society by which they would demonstrate less stress and higher mental wellbeing. Emotion-focussed strategies like being optimistic, avoidance and spirituality, on the other hand, helped the women to adapt to their current circumstances temporarily.

**Table 1.** Socio-demographic characteristics of the participants (*N* = 17).

Variables	*N* (%)
Age	
18–30	6 (35.3)
30–45	11 (64.7)
Marital status	
Married	9 (53)
Separated	3 (17.6)
Divorced	2 (11.8)
Never married	3 (17.6)
Number of children	
None	5 (29.4)
One or more	12 (70.6)
Education	
Non-tertiary qualification	7 (41.2)
Tertiary qualification	10 (58.8)
Employment status	
Voluntary employment	6 (35)
Paid employment	2 (12)
Unemployed	9 (53)
Religion	
Muslim	10 (58.8)
Christian	6 (35.3)
No religion	1 (5.9)

## Social engagement: Problem-solving strategy

Through socialising and being and active member of the society via employment and voluntary work, the participants would develop their networks with people from diverse cultures and improve their integration into the Australian culture and society. Social involvement also improved the participants’ language skills and and gave them a sense of identity.

*Voluntary job, making connections with others give me self-confidence and elevates my spirit. I am cooking for poor people in a charity. . .. So, I am helpful. I see people from different cultures. I can see differences and learn how to behave. . . [also] I cannot learn English at home. I have to talk to people to learn. This work has helped me to get out of the home. (Ava, 32)*

Lack of childcare support particularly for single mothers, however, was a significant challenge that affected their willing and ability to get involved in work market. To overcome this challenge, some joined free play groups to improve their English and socialise with other mothers with similar aged children.

*I take my daughter to park, play groups and mothers’ groups. These help me to socialise and talk to others and get familiar with different cultures. (Nazi, 34)*

Stress of facing a new culture was another barrier that demotivated some participants from seeking social engagement. To avoid being stigmatised due to their socio-cultural differences, some participants decided to quit their cultural/religious practices. One participant from a conservative religion background converted to Christianity to be accepted by the community and be able to engage in social activities.

*After settling in Sydney, I started going to a church for English language classes. I was feeling like a stranger there with scarf on. It was very difficult for me to take off my hijab. . . I was depressed and I didn’t know what is right and what is wrong. . . So I changed my religion and selected Christianity, then I took off my scarf. (Mahsa, 31)*

## Adjusting life plans: Problem-solving strategy

Indefinite insecure residency prevented most participants of pursuing the goals they had previously planned for. To adapt to the current resettlement barriers, some participants changed their life plans and this helped them to maintain their hope and find new opportunities. Enacting a new life plan helped one participant to control her life, avoid idleness and maintain her mental wellbeing.

*I realised that I was not allowed to study and work as an asylum seeker. So, the best thing was to have a baby and extend my family. . . If I could study and work here, I would choose to study first, then have a baby. (Nazi, 34)*

## Seeking support from formal and informal resources: Problem-solving strategy

To regain their lost social networks and socio-economic status, most participants sought support from formal and informal resources. Formal resources included government-funded organisations like counselling services, religious communities and case workers. Friends and family members were informal resources. Despite the ongoing traumatic challenges, the participants in this study were facing post migration, support from available resources meaningfully reduced the adverse psychological outcomes.

*I saw many angels here. When my husband and children left me here [in Australia] alone and returned to Iran, I became familiar with many angels. My landlady, my psychologist, my case worker, and my friends. They all supported me in many ways. Cooking, writing my CV, financially, and psychologically. (Nasrin, 35)*

While the post-migration difficulties were very extensive for this participant, access to informal and formal support resources was perceived as a miracle that could happen only with intervention of extraordinary power from angels. In addition, although she still needed mental health support to deal with the distress of separation from her husband and children, the emotional support from her friends was vital in protecting her from an established and diagnosed mental disorder.

The support is not only emotional support, but formal resources were mainly sought for legal and financial supports. Lack or limited study/work permission placed many participants at a state of poor communication, lack of knowledge and poverty. The role of caseworkers was highlighted by most participants as connecting them to the financial and legal supportive resources.

*My caseworker is doing everything for me. After childbirth, she did all paperwork for my child. She referred me to SSI [Settlement Services International] for getting monetary help. She bought some necessary stuff for my baby. I’ve heard some asylum seekers have no good case workers. I can’t imagine how I could survive without her help. (Hale, 25)*

Sharing experiences and stories with an informal support like a friend or family member was another strategy that reduced the burden of stress.

*I am homesick, but I feel calm when I talk to my sister. She listens to me, my close friend too. When I talk to her [close friend] I feel happy. (Elena, 28)*

Contrary to the above participant, some others minimised their communication with the Iranian community due to stigma around ‘boat arrival people’.

*I can’t ask any help from Iranian people who already settled down in Australia. They will refuse me and judge me as a criminal person. . . If I ask for help, then I must tell them how I came to Australia. (Zahra, 27)*

## Positive thinking and maintaining hope: Emotion-focussed strategy

Focussing on positive aspects of living in the host country as achievements of immigration was another strategy that enhanced the participants’ resilience. Highlighting the positive aspects like freedom and safety boosted the participants’ optimism towards their current and future life in Australia.

*We were shocked at [the] first days of living in the community . . . I am a bit better now because gradually I found out I am safe and they [the Australian Government] care about my child. They care about my health . . . What might happen if I was in Iran with this [sick] child?. (Nazi, 34)*

Maintaining hope and optimism, although did not change their circumstances, helped them strengthen the purpose of their migration and view the negative challenges as tolerable in this instance.

## Avoidance: Emotion-focussed strategy

Participants who found their circumstances out of their control decided to ignore the difficulties via reducing their contacts with their friends and families. Avoidance seems to be helpful for a short term, however, could not protect the participant from psychological issues and long-term need for mental health treatments.

*I like to escape from my problem and don’t think about them. I try to make myself busy with working. . . I try to make myself tired [outside] then just watch movie and sleep at home. . . I don’t contact my parents in Iran to not hear about their problems. I can’t help them. So, why should I call them? (Nasrin, 35)*

## Spirituality: Emotion-focussed strategy

Some participants who found themselves disempowered, unsupported and unable to control their lives, relied on a higher power to protect them against vulnerabilities. Experiencing lower stress through spirituality, on the other hand, would help the women to focus on their available resources and abilities to ease their settlement process.

*I have religious beliefs and pray. I pray every night in bed. . . I believe that God knows something that I don’t. Maybe I get to know them one year later. I keep talking to God and I am sure he will help me one day. (Sima, 39)*

## Discussion

This study explored emotion-focussed and problem-solving strategies that participants applied to enhance their resilience and maintain their mental wellbeing when facing post-migration challenges. Although emotion-focussed strategies could not control or change challenging circumstances, they gave the women a peaceful mind to concentrate on controllable challenges for which they applied problem-solving strategies that empowered them to deal with the ongoing issues.

Due to lack of the ability to work, the women typically engaged in voluntary jobs to establish connections and improve their English language and cultural competency. Through these opportunities, they also developed social skills and felt integrated and accepted in their new country. Previous studies have highlighted positive impacts of participating in social activities and volunteering on learning language, establishing of social network, capital building and provision of emotional and material support (Dako-Gyeke & Adu, 2017; Hanley et al., 2018; Khawaja & Hebbani, 2019; Melamed et al., 2019). Also, this is discussed in the resource-based model for migration (Ryan et al., 2008) that having a developed social network enhances optimism for the future and resilience towards settlement in a culturally diverse community (Ryan et al., 2008). Some challenges are seen as barriers to successful engagement though. Participants with children often experienced isolation and viewed themselves as burdens to the host society (Fleay & Hartley, 2016). Providing childcare support could enable these women to participate in social activities and volunteer positions.

In addition, some participants identified themselves isolated due to their religious practice or Middle Eastern appearance in Australia as a white dominated country. A study on Afghan asylum seekers in European countries highlighted the role of political statements that construct particularly Muslim migrants as threats to national security and culture. Such discourses legitimise dehumanisation and public actions against the new commers by creating concept of *self* and *others* (Sajjad, 2018). To evoke the feeling of belonging to the new society and foster social networking, some of the participants in this study attempted to suppress their cultural and religious identity via converting to Christianity, changing their names or altering other distinguishing attributes. Mirroring the findings of this study, Iranian asylum seekers in Sweden, found religion conversion an opportunity for social structure, connecting to others and sense of inclusion (Öztürk, 2022).

The women reached out to formal resources like their case workers and mental health counsellors to get emotional, financial or legal support. While some acknowledged the support they received from the host community, family and close friends, others found themselves isolated from the Iranian community due to stigma around Iranian people who arrived in Australia by boat. This finding is in contrary to studies on asylum seekers and refugees from different ethnicities (Hanley et al., 2018; Khawaja & Hebbani, 2019). It is stated in the research that being isolated from ethnic community will increase vulnerability of asylum seekers and result in slower integration and exacerbated mental health issues (Strang & Quinn, 2021), while belonging to an ethnic community and being supported by people from the same background would lead to improved mental health (Baird, 2012; Hanley et al., 2018; Nahidi et al., 2018).

A study on Syrian refugees residing in Jordan identified the readjustment of life plans as a problem-solving strategy wherein individuals continually modify their plans to achieve their established goals (Lyngstad, 2015). Adjusting t life plans enables individuals to utilise their available resources efficiently, sustain their hope and improve their quality of life (Lyngstad, 2015). As the finding of this study, despite restrictive policies that hinder asylum seekers from pursuing their desired goals, such as education and employment, those participants who managed to restructure their life plans by introducing new goals and maintaining hope for the future exhibited enhanced resilience and control over their lives.

Avoidance is a strategy to prevent excessive rumination of challenges and reduce the resulting distress (Saadi et al., 2021). In a recent study in the United States (US), asylum seekers demonstrated active avoidance of recalling or discussing specific details from their past as a way of coping with the trauma they endured (Saadi et al., 2021). In contrast, other studies have linked avoidance to distress and mental health issues across different populations, particularly those who have experienced trauma (Barbieri et al., 2021; McVane, 2020). While some participants in the current study appreciated their participation as an opportunity to share their traumatic experiences and reduce their stress, Schock et al. (2016) underlined stressful impacts of recalling traumatic events amongst asylum seekers. Considering contextual factors, it is necessary to empirically examine the applicability of assuming stress-buffering function of avoidance in asylum-seeker populations.

Reflecting on positive achievements and contrasting them with their previous situation in their home country heightened some participants’ optimism and bolstered their resilience when facing post-migration stressors. In line with this finding, Jewish refugees who lost their possessions during migration to France and the US adopted a positive mindset and nurtured their hope for the future (Arendt, 2017). The positive outlook like sense of freedom, gender equality and safety aided them in reconstructing their lives post migration.

Spirituality, whether through belief in a higher power or engagement in religious practices, served as a protective factor for refugees facing challenging conditions (Eid & Diah, 2019). Essentially, when confronted with stressful circumstances such as insecure residency, individuals who surrendered themselves to the situation and placed their trust to a higher power, exhibited a more positive outlook and greater resilience towards ongoing resettlement challenges (Eid & Diah, 2019).

## Limitations of the study

The cross-sectional design limited a comprehensive examination and understanding of long-term impact of coping strategies on individuals' resettlement and mental wellbeing. Nonetheless, the insights into the feasible strategies that Iranian asylum-seeker women applied to enhance their resilience provide a benchmark for relevant institutions to recognise areas that they should work on to protect asylum seekers’ psychological wellbeing.

Recruitment of participants was a challenge in this study for two main reasons. First, they are a hard-to-reach population for a range of reasons; second, those who have experienced severe trauma or have higher levels of mental health issues might actively avoid social interactions that could reduce their visibility (Ibrahim & Sidani, 2014). However, this study successfully employed strategies such as location sampling and snowball sampling to overcome recruitment barriers. It is also important to note that the study findings are limited due to the sampling frame and contextual basis of the setting,

## Conclusion and recommendations

Overall, the study found that emotion-focussed strategies enabled the women to manage their stresses and emotions temporarily, so they could employ problem-solving strategies which could allow them to effectively address the challenges they faced after migration. However, being an asylum seeker, which entails living in a state of uncertainty, could discourage individuals from developing problem-solving strategies to integrate into a new society. Additionally, immigration policies targeting this population and the limited formal and community support hindered their ability to resettle and integration.

The study suggests strategies to ensure strengthened community ties to maximise the social and emotional support asylum seekers receive. Developing and introducing services, such as language classes, leisure and sport programmes and religious practice networks via social media should be considered to ensure sufficient knowledge is communicated.

The literature has suggested that implementing mental health programmes with a focus on emotion-focussed strategies, such as fostering hope for the future and optimism, can be an effective approach in reducing post-traumatic stress disorder and promoting resilience among individuals (Lancaster & Gaede, 2020).

The current study's findings and subsequent discussions recommend some strategies to enhance coping mechanisms, resilience and mental wellbeing among asylum seekers. First, to address stigma surrounding this population, the study suggests reframing the language and using terms such as ‘newcomers’ or ‘protection-seekers’ instead of stigmatised labels in both media coverage and government documents.
